# Critique on “Evaluation of the Presence of Native Valvular Disease in Patients With Atrial Fibrillation Using the EHRA (Evaluated Heartvalves, Rheumatic, or Artificial) Classification”

**DOI:** 10.1002/clc.70207

**Published:** 2025-09-09

**Authors:** Arooha Javed, Syed Muhammad Savez, Syed Muhammad Rayyan

**Affiliations:** ^1^ Dow Medical College Karachi Pakistan; ^2^ Bahria University of Health Sciences Karachi Pakistan; ^3^ Ayub Medical College Abbottabad Pakistan

To the Editor,

1

We read with great interest the article “Evaluation of the Presence of Native Valvular Disease in Patients With Atrial Fibrillation Using the EHRA (Evaluated Heartvalves, Rheumatic, or Artificial) Classification” by Escolar Conesa et al [1]. A critical clinical question is examined by Escolar Conesa et al that: Does a patient with native valvular disease (EHRA‐2) have a different prognosis than a patient without valve involvement (EHRA‐3) in anticoagulated patients with atrial fibrillation (AF)? The authors report significantly higher rates of major bleeding, cardiovascular mortality, heart failure, and MACE in EHRA‐2 patients in a large multicenter cohort (*n* = 1399) with a median follow‐up of approximately 910 days. They also demonstrate that EHRA‐2 status remained an independent predictor following multivariable adjustment [1].

The authors' use of a large, real‐world data set and their publication of incident rates per 100 patient‐years, a clinically valuable statistic, are commendable. Their straightforward explanation of baseline differences and Cox models makes the main point very evident: native valve involvement in AF is not harmless and should be taken into consideration when determining risk and selecting anticoagulation.

The immediate application of these discoveries to routine practice is limited by several challenges. Mild degenerative regurgitation, aortic stenosis, previous biological prosthesis, and other lesions are all grouped under the EHRA‐2 category. These entities are pathophysiologically distinct and probably pose different risks; the observer cannot determine which valve lesions are the main drivers of the signal without lesion‐specific subgroup studies [2]. The study acknowledges the lack of standardized echocardiographic grading. Prognosis and treatment depend on severity (e.g., mild vs. moderate aortic stenosis or regurgitation); combining all native lesions into one category runs the danger of misclassifying clinical risk [1]. EHRA‐2 patients had higher HAS‐BLED and CHA₂DS₂‐VASc scores and were older. The connection may be partially explained by unmeasured confounders (frailty, frail‐functional status, and hemodynamics related to the valves), even if adjusted models partially address this. After substantial correction, previous research indicated that the EHRA‐2 signal attenuated [3]. Sicker patients or those with less access to monitoring may prefer to stay on VKA, which could explain the apparent protective correlation of DOACs vs VKAs. Causal inference would be strengthened by using instrumental variable techniques, inverse probability weighting, or propensity matching [4]. Only baseline measurements of CHA₂DS₂‐VASc and HAS‐BLED were made; events and comorbidity accrual over ~2.5 years may change treatment and risk choices. Updated analysis would more accurately represent clinical practice [1].

Future research should (a) record the kind and severity of echocardiographic lesions, (b) conduct lesion‐specific outcome analyses, (c) compare anticoagulants using propensity/causal approaches, and (d) take time‐updated risk ratings into account. These actions, if verified, would assist in improving anticoagulation thresholds for patients who are currently close to recommended cutoffs, as also emphasized in the 2020 ESC guidelines on atrial fibrillation management [5]. In summary, Escolar Conesa et al. offer a crucial indication that “non‐valvular” AF is diverse: native valvular involvement can identify a minority at increased risk for thrombosis and hemorrhage, necessitating more careful examination and specialized treatment. This interplay between AF and valvular heart disease, and its implications for anticoagulation, has been highlighted in recent reviews [6, 7]. To inform clinical treatment, the work should stimulate prospective, lesion‐stratified research.

## Ethics Statement

The authors have nothing to report.

## Consent

The authors have nothing to report.

## Conflicts of Interest

The authors declare no conflicts of interest.

## Data Availability

The authors have nothing to report.
